# Neurochip3: An Autonomous Multichannel Bidirectional Brain-Computer Interface for Closed-Loop Activity-Dependent Stimulation

**DOI:** 10.3389/fnins.2021.718465

**Published:** 2021-08-19

**Authors:** Larry E. Shupe, Frank P. Miles, Geoff Jones, Richy Yun, Jonathan Mishler, Irene Rembado, R. Logan Murphy, Steve I. Perlmutter, Eberhard E. Fetz

**Affiliations:** ^1^Department of Physiology & Biophysics, University of Washington, Seattle, WA, United States; ^2^Washington National Primate Research Center, University of Washington, Seattle, WA, United States; ^3^Independent Researcher, Seattle, CA, United States; ^4^Department of Bioengineering, University of Washington, Seattle, WA, United States

**Keywords:** brain-computer interface, neuroprosthetics, closed-loop stimulation, neural recording, neural stimulation, neurochip

## Abstract

Toward addressing many neuroprosthetic applications, the Neurochip3 (NC3) is a multichannel bidirectional brain-computer interface that operates autonomously and can support closed-loop activity-dependent stimulation. It consists of four circuit boards populated with off-the-shelf components and is sufficiently compact to be carried on the head of a non-human primate (NHP). NC3 has six main components: (1) an analog front-end with an Intan biophysical signal amplifier (16 differential or 32 single-ended channels) and a 3-axis accelerometer, (2) a digital control system comprised of a Cyclone V FPGA and Atmel SAM4 MCU, (3) a micro SD Card for 128 GB or more storage, (4) a 6-channel differential stimulator with ±60 V compliance, (5) a rechargeable battery pack supporting autonomous operation for up to 24 h and, (6) infrared transceiver and serial ports for communication. The NC3 and earlier versions have been successfully deployed in many closed-loop operations to induce synaptic plasticity and bridge lost biological connections, as well as deliver activity-dependent intracranial reinforcement. These paradigms to strengthen or replace impaired connections have many applications in neuroprosthetics and neurorehabilitation.

## Introduction

Bidirectional brain-computer interfaces (BBCI) can simultaneously record from and stimulate brain sites. Used in a closed-loop configuration, they can deliver activity-dependent stimulation. Two major applications with neuroprosthetic relevance are to bridge lost biological connections and to change the strength of synaptic connections. A third application is to deliver intracranial reinforcement contingent on patterns of neural activity.

We here describe the latest iteration of our so-called “Neurochip” device. It supports multiple channels of recording and stimulation interfaced with onboard computer chips. It remains small and light enough to be carried on the head of a non-human primate (NHP) and has been deployed in many successful scenarios. The previous version, called Neurochip2 (NC2) (Zanos et al., 2011) had three recording channels and was used in multiple studies: (1) NC2 delivered preprogrammed cortical stimuli and recorded the associated LFPs during sleep and waking in a NHP to document state-dependent changes in cortical potentials evoked from cortical stimulation (Richardson and Fetz, 2012). (2) NC2 was used to deliver open-loop paired-pulse stimulation during free behavior in a study of spike-timing dependent plasticity (Seeman et al., 2017). (3) Operating in closed-loop mode, NC2 delivered stimuli during depolarized phases of delta oscillations during slow-wave sleep (SWS) (Rembado et al., 2017). The results of this study suggested that over 17 successive nights of epidural stimulation phase-locked to oscillatory SWS activity facilitated learning of a neuroprosthetic task. (4) NC2 was used to deliver closed-loop stimulation at a rewarding site in nucleus accumbens triggered by action potentials of cortical neurons, to implement operant conditioning of neural activity during free behavior (Eaton et al., 2017). (5) It was also used to induce cortical plasticity when intracortical stimulation was triggered from EMG activity of forelimb muscles (Lucas and Fetz, 2013). (6) In a study demonstrating plasticity in corticospinal synapses NC2 triggered intraspinal stimuli from action potentials of corticomotoneuronal cells during sleep and wake (Nishimura et al., 2013). Consistent with spike-timing-dependent plasticity, effective delays between recorded action potentials and spinal stimuli were below 50 ms; the shortest delays allowed NC2 to activate motoneurons before arrival of the corticospinal spike and demonstrated consequent suppression of the corticospinal connection. (7) In a study demonstrating efficacy in promoting recovery from spinal cord injury in rats, NC2 delivered intraspinal stimuli triggered from EMG of paretic muscles (McPherson et al., 2015). (8) Another study showed that NC2 could be used to bridge lost biological connections. Monkeys whose nerves to wrist muscles were paralyzed could bridge the nerve block by triggering functional electrical stimulation of the paralyzed muscles with bursts of cortical cell activity *via* NC2 (or with lab instrumentation), restoring the ability to volitionally acquire 1D targets (Moritz et al., 2008). These studies all used the NC2, which had only three recording channels. The NC3 significantly enhances the number of channels that can be recorded and stimulated simultaneously.

A number of comparable BBCIs have been proposed and deployed. One such BBCI (Rolston et al., 2009a, b) is a low-cost system combining amplifier head-stages from Triangle Biosystems (TBSI) with custom interface boards for filtering and stimulation control and a desktop computer data collection card. This system is capable of closed-loop stimulation with response latencies as low as 7 ms (Newman et al., 2012), and can record free behavior in rodents through a tether between the head stages and interface boards. A second BBCI (Azin et al., 2011) was developed using a custom fabricated ASIC to construct a head-mounted device for spike-triggered intracortical microstimulation in an ambulatory rat. It was used to electrically stimulate the secondary somatosensory cortex after activity in the rostral forelimb area was detected. Spike detection on each of the four recording channels was performed in real-time with a dual time-amplitude window discriminator. A third BBCI (Zhou et al., 2019) was used for untethered recording of NHP brain signals during sleep. It can stream 96 channels of LFP at 1 kSps over a wireless connection. It can interpolate over short stimulus artifacts to prevent these from activating the threshold spike-detection used to trigger closed-loop stimulation. A fourth system (Deshmukh et al., 2020) demonstrates the use of an implantable device from TBSI to wirelessly record high-bandwidth data and stimulate peripheral nerves. The implant contains a custom ASIC which could potentially be programmed for low-latency stimulation. TBSI has since been incorporated into Multichannel Systems (MCS), which sells head-mountable wireless recording devices that can use threshold spike-detection to trigger closed-loop stimulation. A fifth closed-loop system (Capogrosso et al., 2016) used a head-mounted recording system (Yin et al., 2014) to detect neural patterns of locomotion in motor cortex of monkeys and triggered patterns of epidural electrical stimulation delivered in lumbar spinal cord to support locomotion on a treadmill. The latter four systems used wireless transmission of data, requiring a receiving station positioned within a few meters of the data collection device; to keep triggered-stimulation latency low closed-loop calculations were performed on-device using custom silicon (Azin et al., 2011; Zhou et al., 2019) or an FPGA (Deshmukh et al., 2020). These five systems all had stimulation compliance voltages of ±12 V or below.

The NC3 was developed primarily as a versatile research tool and employs programmable functionality to support a wide range of neurophysiology experiments. Although this generality and off-the-shelf components come at a cost in size, the NC3 is still small enough to be used in free behavior studies with NHPs. Its main advantages are autonomous operation during long periods of unrestrained behavior and sleep, versatile programmability and the ability to deliver short-latency stimuli with high compliance voltage.

## Neurochip3 Architecture

**FIGURE 1:**
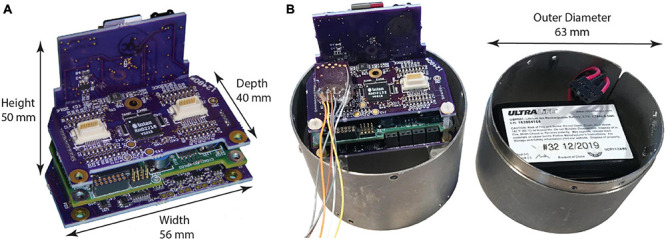
Neurochip3 boards. **(A)** Assembled boards, from top to bottom: input connections and amplifiers chip, MCU and FPGA, and stimulator control; back plane contains power supply, SD Card, IR communications port, and indicator LEDs. **(B)** NC3 with cylinder enclosure, signal input connector, and battery pack in cap.

**FIGURE 2:**
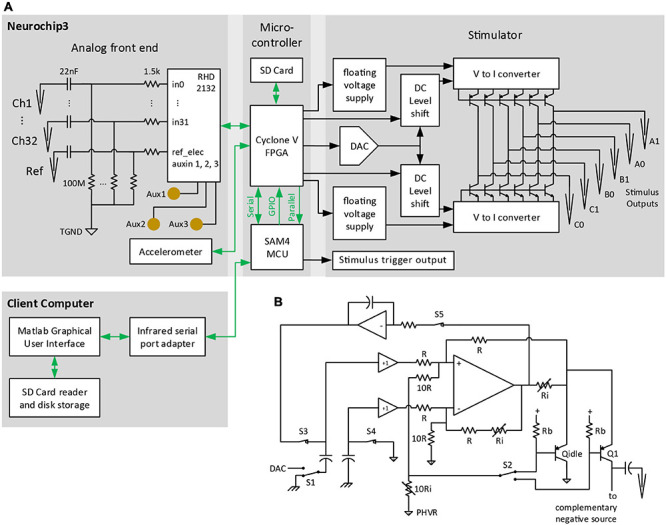
Block diagram of major NC3 components. **(A)** Biophysical analog inputs pass through a 0.07-Hz high pass filter. Data from the analog front end is converted to digital format and delivered to the FPGA. Events from the MCU are relayed over GPIO pins to the FPGA where they can initiate stimulus waveforms previously setup by the MCU over a serial port. The FPGA writes data from the analog front end and MCU to a micro SD Card, and routes selected analog channels to the MCU over a parallel bus. The client computer sends settings to the NC3 over an infrared serial port, and downloads NC3 data from micro SD Cards. TGND, tissue ground; GPIO, general purpose input/output; DAC, digital-to-analog converter. **(B)** Circuit diagram of one of the positive “V to I converter” blocks of the stimulator. PHVR, Positive High-Voltage Return.

Like its predecessors (Table 1), NC3 is designed to operate autonomously within a custom-fabricated titanium casing that is attached to the animal’s skull (Figure 1). The device is powered by rechargeable batteries that can be stored in the casing lid. The NC3 consists of four circuit boards (total of 40 g), powered by two batteries (40 g each), with a total weight of 120 g. NC3 has six main components: the analog front-end, the digital control system, the memory system, the stimulator system, the power converters, and the communication interface (Figure 2A). A Matlab-based graphic user interface (GUI) running on a personal computer is used to upload settings to the NC3 and download and display data. Recordings are stored on a removable flash memory card with capacity of up to 128 GB tested to date. Communication between the computer and NC3 is achieved wirelessly *via* an infrared (IR) data link.

**TABLE 1:** Comparison of Neurochips 1, 2, and 3.

**Feature**	**Neurochip1**	**Neurochip2**	**Neurochip3**
Dimensions (L × W × H)	44 × 20 × 15 mm	55 × 35 × 20 mm	56 × 40 × 50 mm
Power consumption	40–120 mW	284–420 mW	420–630 mW
Recording channels	1 unipolar, 2 bipolar	3 unipolar/bipolar	16 bipolar or 32 unipolar
ADC resolution	8 bit	8 bit	16 bit
Bandpass filter	10 Hz–7.5 kHz	10 or 500 Hz–5 kHz	0.1–2,000 Hz to 0.1–10 kHz
Data storage	8 MB	2 GB	128 GB
Sampling Rate	11 kSps, 2 kSps	10.5/5.3/2.6 kSps	20/10/5 kSps
Stimulation channels	1 unipolar	3 unipolar/bipolar	6 pairable unipolar/bipolar
Compliance voltage	±15 V	±15 V (standard) or ±50 V (high voltage)	±60 V
Operation time	≤60 h	≤48 h	≤24 h

Previous versions of the Neurochip device are Neurochip1 (Mavoori et al., 2005; Jackson et al., 2006a) and Neurochip2 (Jackson et al., 2006b; Zanos et al., 2011). The main differences between the three versions are summarized in Table 1 and described below. The user guide, Matlab interface, MCU and FPGA firmware code, and complete schematics for the NC3 are available online (Neurochip3)^1^ as open source under the MIT license.

### Analog Front-End

The NC3 records biophysical and analog sensor data with an Intan Technologies^2^ RHD2000 series digital electrophysiology interface chip (RHD2216 or RHD2232) based on a low-noise low-power biosignal amplifier design (RHD2000 Amplifier Chips | Intan Technologies). These analog-to-digital data converter (ADC) chips support recording from 16 differential or 32 single ended analog inputs with 16-bit resolution, ±5 mV input range, 2.4 μV RMS noise, and 192× input gain for recording physiological signals. There are also three unamplified auxiliary analog inputs with a 0.1 –2.4 V input range for sensors or other signals. One of these analog inputs can be used to monitor stimulus voltages delivered during the experiment. The Intan RHD2000 chips employ an adjustable bandpass filter (0.1–2,000 Hz low cut, 100–20,000 Hz high cut) applied globally to biophysical channels. The NC3 itself employs a front-end filter (0.07 Hz high pass) on each physiological input channel before relaying the signals to the Intan ADC, and allows sample rates of 5, 10, or 20 kSps individually settable for each channel. Experimenters must select an appropriate bandpass filter for the Intan chip such that the high cut setting is below half the lowest used sample rate. Stimulus artifact reduction is handled by the “fast settle” feature of the Intan amplifier which drives the analog outputs to a baseline “zero” level for a brief period of time (200–3,200 μS) after a stimulus event. Electrode impedance can be tested in a special mode (accessible through the GUI) using the Intan RHD2000 chips’ built-in circuitry for impedance testing.

The NC3 is equipped with a 3-axis accelerometer (STLIS3DH) which is controlled by the FPGA. This records the magnitudes of X, Y, and Z movements, and temperature data at 100 samples per second with a ±2 g range and 10-bit accuracy. This information may be relayed to the microprocessor, allowing events to be triggered from movements.

### Digital Control System (MCU and FPGA)

The NC3 digital control system consists of an Atmel SAM4 ARM Cortex M4 microprocessor and Altera Cyclone V FPGA (5CEBA2 or 5CEBA4). The SAM4 microcontroller unit (MCU) is responsible for communicating with the client interface and controlling the overall experiment progress. The FPGA is responsible for communicating with on-board devices and directing data to the MCU and SD Card storage (Figure 2A). The FPGA is also capable of performing up to eight parallel IIR filters on individual channels, or pairs of ADC data channels, and can rectify or artifact-suppress the filter outputs.

The MCU is a low power 32-bit ARM RISC architecture chip with support for hardware multiply and divide instructions and runs at a clock speed of 48 MHz. It has 256 KB of flash memory for storing program code, and 32 KB of RAM for run-time storage of parameters and variables. This chip has a serial communications port which runs the infrared transceiver to communicate with the client computer, as well as serial and parallel ports for communicating with the FPGA. The MCU handles experiment parameters and setup, and runs the event generators, window discriminators, and stimulus conditions. Event generators may be configured to time periods with fixed or random (uniform or exponentially distributed) intervals, or act as window discriminators to detect when a sampled analog signal crosses a threshold and passes through one or two user-defined windows. These events can be used as acceptance pulses to trigger contingent delivery of electrical stimuli through the stimulator system. The MCU controls progression through up to eight different stimulation conditions; each condition may specify its own set of stimulus waveforms and requirements for triggering. Stimulus waveforms are defined by an initial delay from its trigger, amplitude, width, count, inter-pulse interval, and output pins. Stimulus pulses are bi-phasic, and the phases may be set asymmetrically in both amplitude and width. The output pins may be configured as either single-ended or differential; however only two output pins (anodal and cathodal) may be active simultaneously. Up to six stimulus waveforms for each condition may be specified. If pulses from two or more waveforms would overlap, the pulses are interleaved as time permits. Requirements for triggering stimuli are flexible and users may specify which stimulus waveforms are triggered by which events during specified conditions. Potential triggers may be rejected if event occurrences or source analog levels meet certain requirements, such as counts in a past time interval, or values falling inside or outside data windows.

The FPGA provides low-power configurable logic for parallel operation of communications and computation for all connected devices. It operates at 40 MHz and offers either 25,000 or 49,000 logical elements with 1.9 or 3.4 Mbits of memory, depending on the FPGA version. The FPGA requests and accepts data from the ADC and Accelerometer, controls stimulus waveforms, and routes data to the MCU and SD Card. This frees the MCU from handling very large amounts of data and allows low system clock rates. The flexibility of a programmable logic device such as an FPGA diminishes the need for custom hardware for neural data processing (Sodagar et al., 2007).

### Memory System

Data from the ADC, MCU, and Accelerometer can be stored on a microSD flash memory card with recommended sizes from 32 to 128 GB. The memory card can be manually removed from the NC3 and placed in a USB drive to download the recorded data to a personal computer. The 128 GB memory card is sufficient to hold 24 h of continuous data for 32 channels recorded at 20,000 samples per second. Time stamp data for digital events, condition transition times, stimulus events, and network events are stored interleaved with the digitized analog signals.

### Stimulator System

The stimulator system in NC3 is a direct descendant of the high-voltage Neurochip2 stimulator and delivers constant-current pulses within an output compliance range of ±60 V. Up to 350 stimulation pulses (1 mA amplitude, 0.4 ms duration waveform) per second can be output at this voltage without noticeable sagging. Up to 700 of these pulses can be output per second if the voltage is limited to ±50 V. We have found this range adequate for reaching the 0.5–5 mA threshold for evoking motor output with relatively low impedance electrodes (∼10 kOhm at 1 kHz) placed at the cortical surface, and for delivering 10–500 μA stimuli through higher impedance microelectrodes (up to 100 kOhm) as found in the Utah electrode array (Utah Array).^3^

In contrast to the three bipolar stimulation channels in Neurochip2, each of the six stimulus output pins in NC3 may be differentially paired with any other or used by itself, allowing for up to six bipolar or monopolar stimulus channels available for each of the eight separate stimulation conditions. The high compliance voltage stimulus outputs cannot be multiplexed with the analog inputs as some other technologies allow (Jimbo et al., 2003; Olsson et al., 2005). Each stimulus channel can have a unique set of stimulus parameters, including anodal pin, cathodal pin, current intensity, pulse width, number of pulses per trigger, pulse train frequency, and trigger-to-stimulus latency. All stimulation channels share programmable high-output impedance current sources, precluding simultaneous delivery of pulses on multiple channels. However, switching times between different channels are sufficiently brief (0.4 ms typical) to provide near-simultaneous (0.8 ms leading edge to leading edge delay) and precisely timed sequential stimulation. Maximum pulse frequency depends on stimulation intensity, but for normal amplitudes (<1 mA) the NC3 can output up to 500 stimulation pulses per second. A special high frequency burst mode can be selected by setting the pulse interspike interval to 0. This can produce up to 20 back-to-back pulses within 2 ms, achieving 10 KHz pulse trains several times per second.

The NC3 stimulator is implemented with two modified Howland current sources driving high-voltage bipolar transistors to minimize power consumption (Figure 2B). Essentially all of the quiescent current consumed flows within the floating 4 V supplies, except while delivering a stimulus pulse. At idle, the integrator associated with S3 and S5 feedback sets the output bias current to ∼100 nA through pseudo-output transistor Q_*idle*_ into the floating positive ground (Figure 2B), compensating for any offset voltage in the Howland op amp. The only high-voltage current loss is the <50 nA Icbo of each output transistor. To deliver a positive current pulse, switches S3-5 open, S2 is set to the desired output transistor, and S1 is switched to the DAC output voltage. The capacitors couple the DAC voltage level to the Howland sub circuit, which ensures that the same voltage appears across the output current-setting resistor Ri connected to Q1’s emitter. 0.1% resistors are used to ensure accurate matching with the complementary negative source in delivering biphasic output pulses. The low-impedance, common-base input ensures that the Howland circuit will be stable even without trimming the resistor network. Transistor base current is corrected by feedback to the Howland circuit, ensuring accurate current output and increasing the already high output resistance. MOSFET output transistors were not used since they require larger floating supply voltages, have high output capacitance (diverting output current from the electrodes), and have poorly specified leakage currents approaching minimum stimulation levels. The output impedance of this circuit is limited primarily by the output transistor and wiring capacitance.

The NC3 can monitor the output voltage from one of the stimulator channels. This allows the impedance of the electrode to be monitored over the duration of the experiment and allows verification that the target current was consistently delivered.

### Power

**FIGURE 3:**
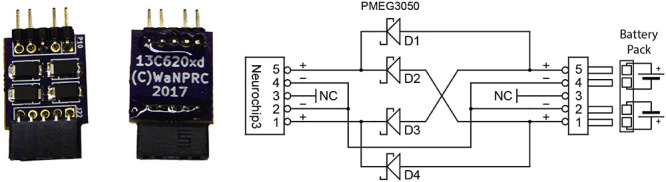
Optional battery combiner circuit uses four Schottky barrier rectifiers to allow full discharge of both batteries at only a small loss in total power delivered to the NC3.

The electronic circuits are powered by two rechargeable, 3.7 V lithium-ion batteries each with 1.75 Ah capacity (UBP103450, Ultralife Batteries). 3.3, 2.5, and 1.1 V DC power supplies for the MCU, FPGA, and other digital components are generated by a triple buck-boost DC/DC power converter (LTC3521). The NC3 uses a Texas Instruments TPS61045 with a CoilCraft HP1-0059 transformer to create the floating 4 V supplies, and a Maxim 1771 to generate the ±15–55 V supply for the stimulator. The standard battery configuration uses one battery for the digital components and the other for the stimulator power supply to provide noise isolation. However, the two batteries may be combined with a small circuit board (Figure 3) to provide longer running times since the stimulator power draw is much less than that of the digital components. Power consumption as measured by a multimeter is approximately 420 mW at idle, 480 mW when sampling all channels at the highest sampling rate, and 630 mW when recording all channels to the SD Card. Stimulation pulses consume additional power which varies with stimulation rate, electrode impedance, and pulse amplitude and width. Moderate amounts of stimulation (1 mA amplitude, 0.4 ms duration waveforms) require about 10 mW (10 Hz stimulation) to 40 mW (100 Hz stimulation) of additional power. New (fully charged) batteries typically yield run times from 12 h (all channels, 20 kSps, standard battery configuration) up to 23 h (all channels, 20 kSps, 10 Hz stimulation, combined batteries). A small amount of power can be saved by reducing channel count and sampling rates, but this extends run times by only 15–30 min. If stimulation is not required, turning off the stimulator power completely can also extend run times by a similar small amount. Older batteries that have been used weekly for several years can still produce 10 h run times with the standard battery configuration.

The temperature of the NC3 circuit boards were measured at 30°C with a FLIR thermal imager while the NC3 operated at high power consumption. Hot spots such as the FPGA and power components measured at most 38°C, well within normal body temperature. Thus, the NC3 should not exceed body temperature when used in a head-mounted container.

### Interface

**FIGURE 4:**
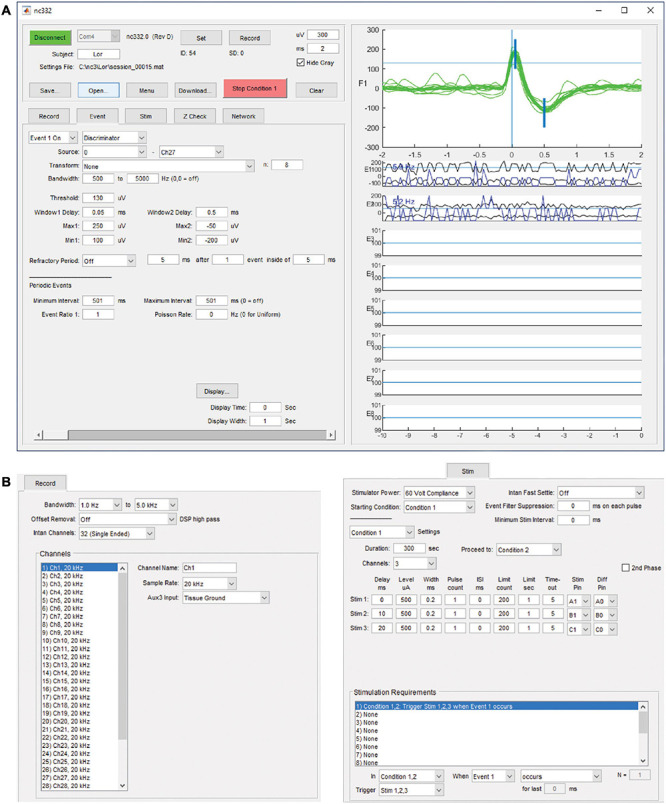
Matlab-based graphical user interface on a PC. **(A)** Upper left panel contains controls for interacting with the NC3. Lower left tabbed panel contains settings for recording, event generation, spike discrimination, stimulation, impedance testing, and neural network simulation. Right panel shows data sweeps and summaries for checking performance and setting up correct event generation. It can also be used to review previously collected data. **(B)** Tabbed panels for record and stimulation settings.

Communication and interfacing are handled by an application written with Matlab (Mathworks, Inc.), which sends and receives information over a serial-to-infrared adapter link to the infrared transceiver on the NC3. While this link is active, parameters may be downloaded, data recording may be started or halted, and summary data from on-going collection will be displayed. The summary data includes event occurrences and data envelopes for each of the eight event generators, stimulus occurrences for the six stimulus outputs, and data sweeps from the source channel of the selected event generator (Figure 4A). The data sweeps can be used to check recording settings, set up window discriminators, and examine stimulus responses and artifacts. An external stimulus trigger output from the MCU is accessible on a front connector pin and can be used to trigger other devices, for example a light-emitting diode for optical stimulation. There are eight GPIO pins available from the FPGA for testing or adding external peripherals. These pins have been used in some projects to connect the NC3 to wireless devices. Figure 4 illustrates the graphical user interface as seen on a PC.

### Firmware

Both the MCU and FPGA are controlled by resident firmware programs downloaded through the JTAG programming port located at the front-left of the middle circuit board. The firmware for the MCU is written in the C programming language using a code project managed by Atmel Studio (v6.2). The resulting compiled binary image is stored in the MCU’s flash memory and executes directly on power-up. The firmware for the FPGA is maintained as an Intel Quartus Prime (v18.1) project and is written primarily in VHDL. Its binary image is stored in an EPROM chip and is transferred to the FPGA on power-up for execution. On power-up, both devices initialize their respective hardware and then run a continuous process until power is removed. The MCU will receive and execute commands given by the client computer over the infrared transceiver, and the FPGA will receive and execute commands from the MCU over a serial port. This allows the client computer to issue high-level commands such as “Use these parameters” or “Start recording,” while the MCU handles configuration and decision making, and the FPGA handles low-level timing, control of connected devices, and bulk data processing.

### Neural Network Simulation

**FIGURE 5:**
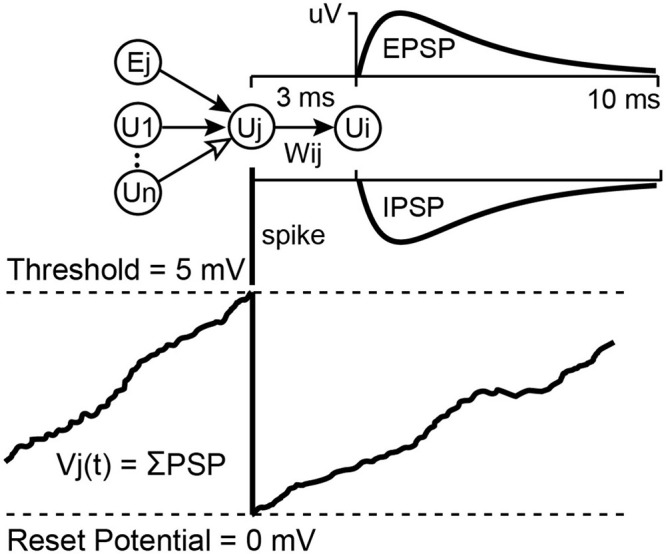
Unit potential calculation for the integrate-and-fire neural network. Spiking input from connected units sum into a target unit’s potential Vj(t). If Vj(t) reaches threshold it is reset to 0 and the unit sends a spike to all of its target units, which will cause a Post Synaptic Potential proportional to Wij. These PSPs may be excitatory or inhibitory, depending on the sign of Wij (for more details see Shupe and Fetz, 2021).

The NC3 can run an integrate-and-fire spiking neural network (SNN) on the FPGA in parallel with its other operations. Details of the SSN are described elsewhere (Shupe and Fetz, 2021). This feature can be used to process multiple inputs and generate events to trigger stimuli. The SNN may contain up to 256 units and as many as 2,000 connection weights between these units. The first eight units are inputs and outputs which can receive input from and relay spiking activity to the NC3 event generators. The hidden SNN units receive input spikes from other units or from the randomly generated bias spikes which every unit receives as an independent spike stream. The network runs at 10,000 updates per second. On each update, the SNN integrates recent spiking activity to each unit. This integrated activity is intended to represent the summed post-synaptic potentials (PSPs) of biological neurons (Figure 5). When a unit’s activity exceeds a threshold θ, its activity is reset to zero, and an output spike is initiated from that source unit to all of its target units. Connections between units are defined by the amplitude (in μV) of a PSP shape defined by the difference between two leaky integrators.

The SNN is implemented as a single state machine on the FPGA with access to a list of unit potentials, a list of delayed-spike queues, a list of weights connecting units, and a set of global parameters affecting all units. The global parameters include spike conduction delay (3.5 ms max), a fast and a slow PSP decay constant, bias input chance, bias strength, and unit firing threshold. Each unit maintains a fast and a slow decaying unit potential and has its own list of connections from which it receives input. These connection lists contain a source unit index and a weight value for each incoming connection. In the Matlab interface source unit indexes –1 to –8 indicate spike inputs taken directly from the eight event generators which are currently the only external inputs to the SNN. Firing threshold, connection strengths, and unit potentials are 16-bit signed values designated in microvolts. Decay constants and the bias chance are 0.16-bit fixed point values representing floating point values between 0.0 and 1.0.

At each 0.1 ms time step, the fast and slow decay unit potentials are multiplied by their respective decay constants, keeping the most significant 16 bits. The state machine then examines the delayed spikes from each unit’s sources. If a source spike occurred, then the connection weight from that source is added to the destination unit’s fast and slow unit potentials. Then each unit is checked for a possible bias spike addition by comparing the bias chance against a pseudo random value selected for each comparison. At this point, units with a difference between their slow and fast potentials exceeding threshold will initiate a spike in their respective spike queue and have their potentials set to zero. Output spikes from the first 16 units may be routed to the MCU and will be saved to the SD Card during recording. Currently the MCU can use the first eight of these to activate one of the eight event generators so that SNN outputs can trigger stimulation.

## Stimulus Artifacts

**FIGURE 6:**
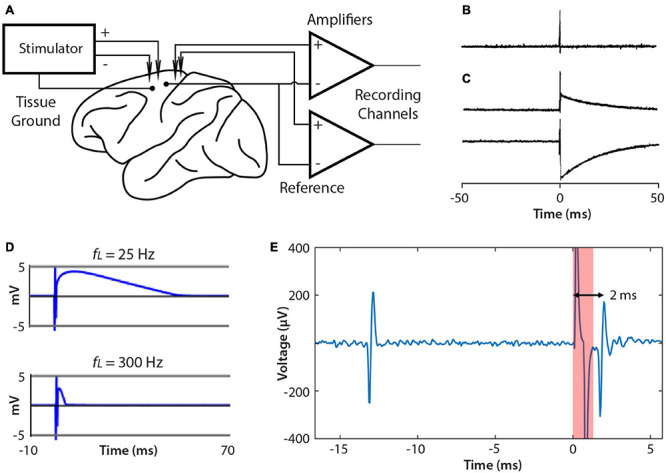
Connections to brain and artifacts. **(A)** Single ended recording uses the same reference site for each channel. The NC3 stimulator employs a tissue ground and supports differential stimulation, which can reduce artifacts. **(B)** Stimulus artifacts that do not exceed the amplifier input range are very brief (<2 ms). **(C)** Examples of stimulus artifacts that may occur when stimulation saturates the input amplifier. **(D)** Artifacts are affected by the input filter’s low pass cut-off (*fL*). Increasing *fL* from 25 to 300 Hz shortens the artifact duration. **(E)** Example of a spontaneous spike (left) followed by a stimulus-evoked spike (right).

A significant issue when BBCIs stimulate and record simultaneously is the size of the stimulus artifact, which appears in the recording. Figure 6A shows the typical relation of the leads from NC3 amplifiers and stimulator to the animal. When delivering a large stimulus, the NC3 recording channels can show a stimulus artifact during which reliable signal recording is compromised. The recorded artifact is brief (<2 ms) when the stimulus is sufficiently low that the input does not exceed the ±5 mV input range of the amplifiers (Figures 6B,E). Prolonged artifacts occur when they exceed the input range of the amplifiers and when recording low-frequency signals (Figure 6C). Under these conditions long decaying artifacts occur with a time course roughly proportional to the inverse of the high pass filter setting (i.e., the low cut-off frequency of the bandpass filter) (Figure 6D). For example, if the Intan filter bandwidth is set to 10 –5,000 Hz, the recovery time can be as long as 1/10 Hz = 0.1 s. For the frequency bands used to discriminate neural action potentials (500 Hz–5 kHz) the artifact is sufficiently brief to reliably record within a ms after the stimulus (Figure 6E).

Several methods have been proposed to reduce stimulus artifacts (Brown et al., 2008; Rolston et al., 2009a, b; Uehlin et al., 2020). The NC3 can utilize the “fast settle” feature of the Intan amplifier to help reduce artifacts, although in practice transiently switching this function can cause a similar long-term artifact. The artifact can also be reduced by using differential stimulation between electrodes placed as close to each other as possible, and by separating the tissue ground and signal reference (Figure 6A).

### *In vivo* Testing

The features of NC3 have been useful in several *in vivo* studies, largely unpublished to date.

### Multiple Closed Loops

**FIGURE 7:**
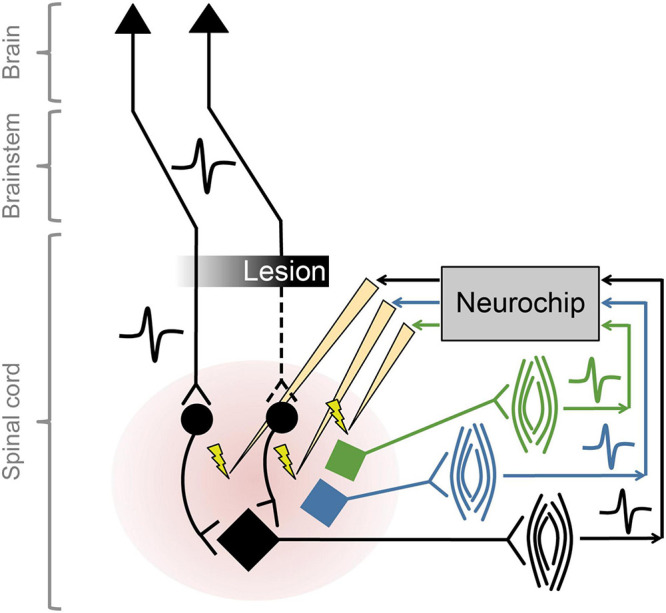
Multiple simultaneous closed-loop stimulation with NC3. As shown for the black circuit, descending pathways are partially lesioned by spinal cord injury. The paretic black muscle generates motor unit potentials that are converted to stimuli delivered to spinal neurons in the pathways to the black motoneuron (at intensities subthreshold for evoking movement). Comparable circuitry pertains to the blue and green motoneurons.

The NC2 was used previously to improve motor function in rats with cervical spinal cord injury by implementing activity-dependent stimulation of the spinal cord (McPherson et al., 2015), while control open-loop stimulation had no effect on recovery. Intraspinal stimulation at one spinal site was triggered from electromyographic activity (EMG) recorded from one paretic forelimb muscle. For these studies with rodents the Neurochip is mounted above a behavioral arena and connected to the animal with a flexible cable that does not restrict mobility. We recently expanded the therapy to target multiple neural circuits, exploiting the capability of the NC3 to implement closed-loop stimulation in multiple channels in parallel. The so-called multi-channel, targeted, activity-dependent spinal stimulation (mTADSS) pairs EMG from three muscles with stimulation at three spinal sites. The pairs are chosen to target the primary impairments produced by the spinal cord injury: inability to extend the elbow and the wrist and digits, and weak grip strength. For example, motor unit action potentials from triceps are discriminated by a dual time-amplitude window running on the NC3 MCU; discriminator events then trigger stimulation at a spinal site from which elbow extension is evoked with larger stimulation currents. The three closed loops run simultaneously and independently to induce plasticity in separate spinal pathways (Figure 7). To prevent stimulus-evoked responses in any muscle from triggering additional stimuli, the NC3 imposes a global refractory period of 10 ms after each stimulus during which no other stimuli can be delivered. We hypothesize that mTADSS activates spinal neurons in specific output pathways coincident with the volitional activation of descending axons involved in controlling the same muscles. The repeated synchronous activation of pre- and post-synaptic elements acts to strengthen the connections between them, leading to recovery of volitional movement. Preliminary evidence indicates that 14 weeks of mTADSS combined with behavioral retraining improved reaching and grasping movements that lasted well beyond the end of treatment (Murphy and Perlmutter, unpublished).

### Cortical Stimulation

**FIGURE 8:**
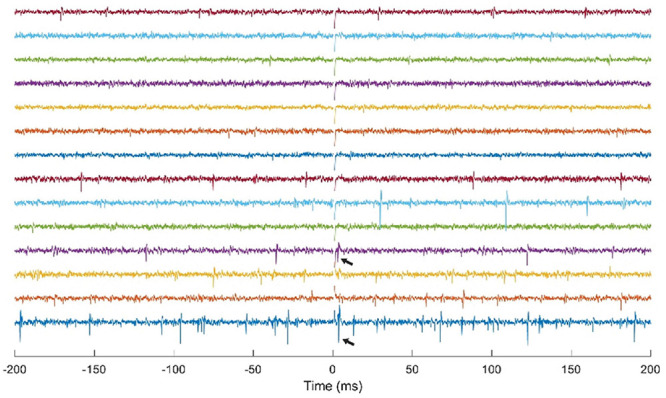
Simultaneous stimulation and multichannel recording of neural action potentials with NC3. Stimulus occurs at *t* = 0. Arrows indicate action potentials evoked by a 15 μA stimulus delivered through a neighboring electrode.

The NC3 has been used for multichannel recording and stimulation in motor cortex of NHPs using a Utah microelectrode array. Though neural responses to stimuli have been previously documented (Butovas and Schwarz, 2003; Tehovnik et al., 2006), they have typically been examined in anesthetized or restrained subjects. One study used the NC3 to investigate the responses of cortical neurons to microstimulation delivered at neighboring sites in freely behaving NHPs over time (Yun and Mishler, unpublished). Figure 8 shows simultaneous traces of neural activity recorded at 14 of 30 sites. The NC3 was programmed to deliver repetitive microstimuli of 15 μA to a site that evoked neural responses at two other sites. Note that the stimulus artifacts (at *t* = 0) are sufficiently brief to allow detection of action potentials within 1 ms, including spikes evoked by the stimulus (arrows). Signals were recorded with a bandwidth of 0.1 Hz–5 kHz and bandpass filtered between 900 Hz and 2 kHz for the figure. Single unit responses to stimuli consisted of a fast excitatory response occurring between 1–10 ms and/or an inhibitory period lasting for up to 100 ms (Yun and Mishler, unpublished).

### Overnight Recording and Stimulation

**FIGURE 9:**
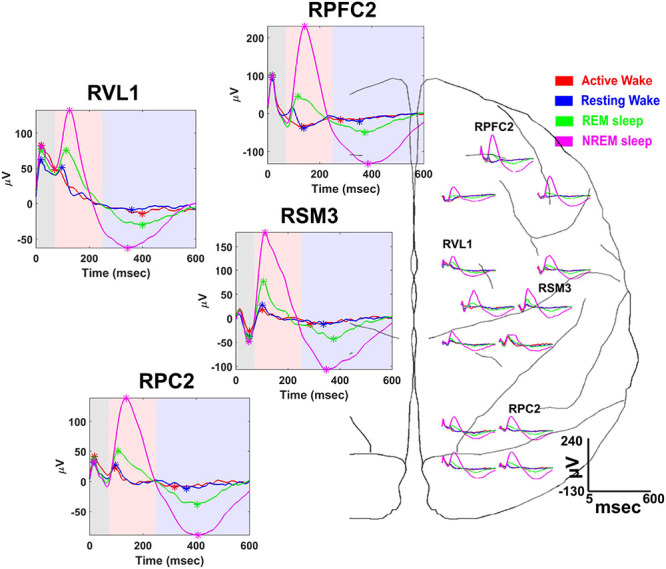
Vagal evoked potentials (VEPs) elicited during different behavioral states: (active-wake AW, red); resting-wake (RW, blue); rapid eye movement sleep (REM, green); and non-REM sleep (NREM, magenta). Representative averaged evoked potentials recorded from the following cortical sites are expanded at left: right prefrontal cortex (RPFC2), right ventrolateral nucleus of thalamus (RVL1), right somatosensory-motor cortex (RSM3), and right parietal cortex (RPC2). Time is measured from the first pulse of the 300 Hz train of 5 pulses delivered to the vagus nerve; colored areas designate the time range for specific VEP components (from Rembado et al., 2021).

A useful feature of the NC3 is its capacity for autonomous operation for long periods of time, providing continuous functionality during different behavioral states such as sleep and waking states. This capability was exploited in a study to examine state-dependence of vagally evoked cortical potentials (VEPs) in response to stimulation of the vagus nerve (Rembado et al., 2021). NC3 recorded intracortical potentials at 16 sites located over the prefrontal, sensorimotor and parietal cortical areas while stimulating the vagus nerve through a bipolar nerve cuff (Cyberonics, LivaNova Inc.). NC3 was programmed to deliver trains of 200 μs biphasic, symmetric current pulses at an intensity between 1,000 and 1,500 μA. Each train consisted of five pulses of different pulsing frequencies ranging from 5 to 300 Hz with 10-s interval between consecutive trains. Figure 9 illustrates the VEPs recorded at multiple cortical sites during four behavioral states: active-wake, resting-wake, rapid-eye movement (REM) and non-REM sleep. The states were defined by spectral characteristics of the cortical field potentials and the accelerometer signal of the NC3.

**FIGURE 10:**
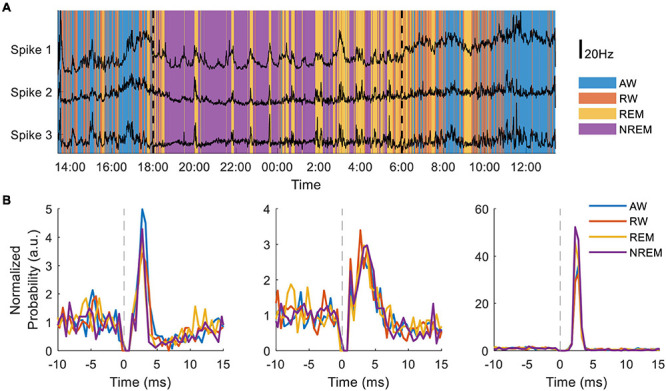
Long-term recording of multiple neurons during sleep and wake. **(A)** Neural firing rates of three neurons recorded for 30 h during four putative behavioral states: active-wake (AW, blue); resting-wake (RW, orange); rapid eye movement sleep (REM, yellow); and non-REM sleep (NREM, purple). During the time between the two vertical dashed lines the animal room light was turned off (18:00–6:00). **(B)** Peristimulus time histograms normalized to average firing rate of three different neurons during the four classified states.

In separate experiments the NC3 has been used to compare state-dependent responses of single neurons to cortical stimulation. Dimensionality reduction using 10-second bins of 16-channel LFP spectral power and the accelerometer recordings from the NC3 were used to distinguish the four behavioral states throughout a 24-h period. The state classifications were further validated by simultaneous movement tracking, electrooculography recordings, and corresponding changes in neural firing. Preliminary evidence indicates that the firing rates of three neurons covaried with each other in a state-dependent manner (Figure 10A). NC3 was used to evoke action potentials from stimuli delivered at a neighboring site (as in section “Cortical Stimulation” above) throughout wake and sleep, and the strength of stimulus-evoked responses varied between states in a manner that depends on the specific neuron (Figure 10B) (Yun, unpublished). The ability of the NC3 for autonomous long-term stimulation and recording can provide further insights into the neural mechanisms operating during sleep, including learning and memory.

### Spiking Neural Networks

**FIGURE 11:**
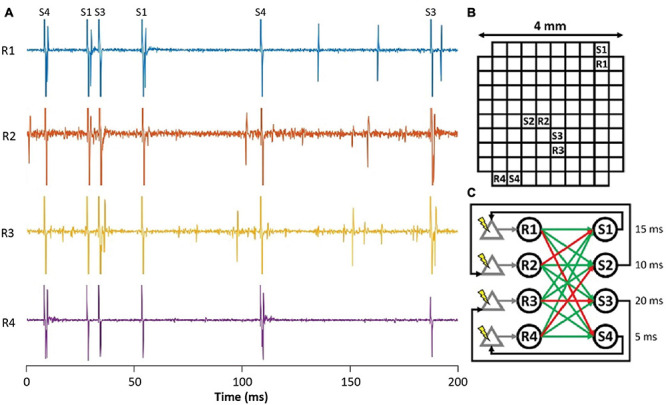
Implementation of a simple closed-loop BCI *via* an ANN. **(A)** Spike activity of simultaneously recorded input neurons (R1-R4) during stimuli delivered at outputs (S1-S4). Labels at top identify stimulus channels producing corresponding stimulus artifacts. **(B)** Location of recording and stimulating sites on Utah array. **(C)** Connectivity of spiking SNN showing excitatory (green) and inhibitory (red) connections from inputs (R1–R4) to outputs (S1–S4). Numbers are delays between output spikes and stimuli.

To investigate closed-loop brain-computer interfaces *via* artificial SNNs, the FPGA on the NC3 was programmed to emulate an integrate-and-fire SNN. Figure 11A shows simultaneous traces of neural activity recorded when NC3 implemented the illustrated SNN. The simple two-layer feed-forward SNN (Figure 11C) received both excitatory and inhibitory spiking inputs from four cortical units (R1-R4). When the membrane potentials of the artificial units exceeded a predetermined threshold, they generated spikes at one of four outputs (S1-S4), and these triggered stimuli delivered at the indicated delays. The stimuli could evoke single action potentials in the input cortical units which created artificial connections between the cortical neurons and altered their typical firing patterns. The locations of recording and stimulated sites in the Utah array are shown in Figure 11B. Note that due to the fast settle feature, the stimulus artifacts were sufficiently brief to produce negligible interference with recorded and evoked spikes (Figures 11, 6E). In the experiments performed to date, the weights and delays of the SNN were arbitrarily chosen to demonstrate its closed-loop operation; future implementations could vary these parameters in user-defined ways (Mishler, unpublished).

## Discussion

NC3 has significantly greater functionality than NC2 (Table 1) and the two have successfully been deployed in many closed- and open-loop applications, described in the introduction and results.

The NC3 can be compared with several other bidirectional BCIs that have been described in the literature (Olsson et al., 2005; Rolston et al., 2009a, b; Azin et al., 2011; Capogrosso et al., 2016; Su et al., 2016; Liu et al., 2017; Zhou et al., 2019); the features of five are summarized in Table 2. The various designs differ in their capabilities and related trade-offs. The NC3 was developed primarily as a research tool with a wide range of capabilities, including a large number of channels all recordable at high sampling rates, and a high-voltage stimulator to deliver controlled current through moderately high impedance electrodes. In comparison a commercially available system from MCS offers similar recording capabilities in a smaller form factor but with lower run time and stimulation power (Wireless-Systems).^4^ Custom ASIC devices (Azin et al., 2011) can be many times smaller than the NC3, but at the expense of the number of recording channels and flexibility in software programmability and setup. One such ASIC (Liu et al., 2017) includes 16 recording channels each switchable with a multi-mode stimulator, and hardware implementations of stimulus artifact suppression, neural feature detection and PID controllers enabling low-power closed-loop operations. Other devices target more specific applications such as the implantable design proposed by Su et al. (2016) which uses a similar biophysical amplifier to the NC3, but cannot match the amount of recordable data or flexibility in programmable stimulation. These systems use wireless communication protocols to transmit data to a base station, while the NC3 uses a micro SD Card for removable data storage. Advancements in wireless technology (Chestek et al., 2009; Rizk et al., 2009; Mirbozorgi et al., 2016; Shon et al., 2018) allow for continuous monitoring of many channels of data, while a removable storage card allows for recording large amounts of data in situations where wireless transmission is impractical. On-board computation supports the ability to deliver short-latency stimuli, which is critical for spike timing-dependent plasticity applications requiring delays below 50 ms. The NC3 uses a commercially available ARM microprocessor and FPGA which give it both ease of programming and high data processing and throughput. This functionality comes at the expense of larger power consumption, physical size, and weight as compared to other systems, but provides a considerable amount of flexibility in experimental setup.

**TABLE 2:** Comparison of NC3 to five other systems.

	**NC3**	**Multichannel Systems (W2100)**	**Azin et al., 2011**	**Su et al., 2016**	**Liu et al., 2017**	**Zhou et al., 2019 (WAND)**
Fabrication	Custom PCBs with commercial components	Commercially available system	Custom ASIC (350 nm CMOS)	Custom PCB with commercial components	Custom ASIC (180 nm CMOS)	Custom ASIC (180 nm), commercial FPGA and radio SoC
Recording channels	16 differential or 32 single ended	4, 8, 16, or 32 per headstage	4	32 unipolar/bipolar	16 single ended	128
ADC resolution	16 bits	16 bits	10 bits	16 bits	10 bits	15 bits
Sampling rate (per channel)	5, 10, or 20 kSps	20 kSps (32 channels), 25 kSps (16 channels)	35.7 kSps	30 kSps	1 MSps aggregate	1 kSps
On board data storage	Up to 128 GB Micro SD Card	None	None	None	None	None
Gain	45 dB	40 dB	60 dB	45 dB	40 dB	0 dB
Bandwidth	0.1 Hz–10 kHz	0.1 Hz–5 kHz	1.1–525 Hz low cutoff, and 5.1–12 kHz high cutoff	0.1 Hz–20 kHz	0.3 Hz–7 kHz	DC-coupled
Input voltage range	±5 mV	±12.4 mV	±0.75 mV (at 60 dB gain)	±5 mV	±9 mV	±50 mV
Input noise	3 μV rms	1.9 μV rms	3 μV rms	3 μV rms	4.57 μV rms	1.6 μV rms
Communication	IrDA, Micro SD Card	Wireless (2.4 GHz)	Wireless 500 kb/s (FSK at 433 MHz)	Enhanced ShockBurst (2.4 GHz GFSK)	Bluetooth	Bluetooth
Communication range	1 m, SD Card (offline (data download)	5 m	2 m			2 m
Battery supply	3.7 V (1,750 mAh) Li-ion rechargeable (×2)	3.7 V (300 mAh) Li-polymer rechargeable (×1)	1.55 V (26 mAh) silver-oxide coin (×1)	3.7 V (200 mAh) Li-ion (×1) with wireless ultrasonic recharge	150 mAh lithium polymer battery with wireless inductive charging	250 mAh lithium-ion rechargeable (×2)
Battery run time	10–24 h	2–6 h	24 h	10 h	24 h	11.3 h
Power consumption	420–630 mW		0.42 mW	15–60 mW	10 mW	172 mW
Size	56 × 40 × 50 mm	15.5 × 15.5 × 6.7 mm	36 × 13 × 6 mm (1.7 g)	35 mm in diameter, 10 mm in thickness	30.1 × 18.3 mm	36 × 33 × 15 mm
Stimulation channels	6 pairable unipolar/bipolar (asymmetric allowed)	2 single ended bipolar (or 2 optical)	4 monophasic or charge-balanced asymmetric biphasic	4 unipolar/bipolar	16 outputs with 4 drivers, biphasic bipolar (asymmetric allowed)	128 outputs with 8 drivers biphasic bipolar (asymmetric allowed)
Stimulation voltage	60 V	10 V	5 V	20 V	5 V	12 V
Current intensity	5 μA–10 mA	Up to 500 μA (1 mA for optical)	0–94.5 μA anodic, and 31.5 μA cathodic	40 μA	20 μA–4 mA	20 μA–5 mA
Pulse width (per phase)	0.01–10 ms	0.1–10 ms	Up to 240 μs anodic, 720 μs cathodic	1 μs minimum	1–255 μs	15–500 μs
Pulse frequency	Up to 500 Hz single pulse, 10,000 Hz short burst			250 Hz	100–1,500 Hz	15–255 Hz
Real-time spike discrimination	Dual time-amplitude window discriminators	Threshold detection	Dual time-amplitude window discriminators	None	Dual time-amplitude window discriminators	Spectal power threshold detection
Accelerometer	Yes	Yes	No	No	Yes	Yes
Gyroscope	No	Yes	No	No	No	Yes
User interface	Matlab	Windows 8.1/10 applications	LabView	Python	Matlab	Python

### Future Features

NC3 can be readily modified to support optogenetic stimulation. In this configuration the stimulator would trigger LEDs that activate light-sensitive excitatory or inhibitory channels in genetically modified neurons. The advantage of optogenetic stimulation for closed-loop applications is the absence of an electrical artifact, the ability to deliver brief activity-dependent inhibitory events and to target stimulation to specific cell types. Neural network simulations predict that spike-triggered inhibitory stimulation could lead to decreases in the strength of synaptic connections (Shupe and Fetz, 2021).

Another future enhancement would be greater capability for wireless communication (Rosenthal and Reynolds, 2019). This would enable streaming large amounts of data to offline receivers, which would facilitate uninterrupted long-term recording. On the other hand, the storage to an onboard memory card has the advantage of circumventing the challenges of establishing reliable wireless transmission.

There is currently substantial space available on the FPGA for more parallel computations. This could include automated spike detection on all channels, computing Fourier transforms of analog data, off-loading computation currently performed by the MCU, and implementing a stimulus artifact cancelation algorithm. With the programmability of the FPGA, these features come only at the expense of software development and a minor increase in the NC3’s power requirements.

Future designs of the Neurochip will be dependent on advances in technology and be driven by a wide range of BBCI applications. Given the ability to incorporate future commercial components, subsequent versions of the Neurochip will empower new and more advanced functions for both research and neuroprosthetic applications.

## Data Availability Statement

The user guide, Matlab interface, MCU and FPGA firmware code, and schematics for the NC3 are available online (https://depts.washington.edu/fetzweb/neurochip3.html) as open-source under the MIT license. Further inquiries can be directed to the corresponding authors.

## Ethics Statement

The animal study was reviewed and approved by the University of Washington IACUC.

## Author Contributions

LS and EF wrote the manuscript. LS developed the NC3 firmware and software. FM designed the NC3 stimulator and printed the circuit boards. GJ developed the FPGA firmware. RY, JM, IR, RM, and SP performed the designated research with NC3. All authors contributed to the article and approved the submitted version.

## Conflict of Interest

The authors declare that the research was conducted in the absence of any commercial or financial relationships that could be construed as a potential conflict of interest.

## Publisher’s Note

All claims expressed in this article are solely those of the authors and do not necessarily represent those of their affiliated organizations, or those of the publisher, the editors and the reviewers. Any product that may be evaluated in this article, or claim that may be made by its manufacturer, is not guaranteed or endorsed by the publisher.

## Footnotes

1
https://depts.washington.edu/fetzweb/neurochip3.html

2
https://www.intantech.com

3
https://www.blackrockmicro.com

4
www.multichannelsystems.com

## References

[B1] Azin M. Guggenmos D. J. Barbay S. Nudo R. J. Mohseni P. (2011). A miniaturized system for spike-triggered intracortical microstimulation in an ambulatory rat. *IEEE Trans. Biomed. Eng.* 58 2589–2597. 10.1109/tbme.2011.2159603 21690007

[B2] Brown E. A. Ross J. D. Blum R. A. Nam Y. Wheeler B. C. DeWeerth S. P. (2008). Stimulus-artifact elimination in a multi-electrode system. *IEEE Trans. Biomed. Circuit. Sys.* 2 10–21. 10.1109/tbcas.2008.918285 23852629

[B3] Butovas S. Schwarz C. (2003). Spatiotemporal effects of microstimulation in rat neocortex: a parametric study using multielectrode recordings. *J. Neurophysiol*. 90 3024–3039. 12878710 10.1152/jn.00245.2003

[B4] Capogrosso M. Milekovic T. Borton D. Wagner F. Moraud E. M. Mignardot J. B. (2016). A brain-spine interface alleviating gait deficits after spinal cord injury in primates. *Nature* 539 284–288. 10.1038/nature20118 27830790 PMC5108412

[B5] Chestek C. A. Gilja V. Nuyujukian P. Kier R. J. Solzbacher F. Ryu S. I. (2009). HermesC: low-power wireless neural recording system for freely moving primates. *IEEE Trans. Neural. Syst. Rehabil. Eng.* 17 330–338. 10.1109/tnsre.2009.2023293 19497829

[B6] Deshmukh A. Brown L. Barbe M. F. Braverman A. S. Tiwari E. Hobson L. (2020). Fully implantable neural recording and stimulation interfaces: peripheral nerve interface applications. *J. Neurosci. Methods* 333:108562. 10.1016/j.jneumeth.2019.108562 31862376

[B7] Eaton R. W. Libey T. Fetz E. E. (2017). Operant conditioning of neural activity in freely behaving monkeys with intracranial reinforcement. *J. Neurophysiol.* 117 1112–1125. 10.1152/jn.00423.2016 28031396 PMC5340878

[B8] Jackson A. Mavoori J. Fetz E. E. (2006a). Long-term motor cortex plasticity induced by an electronic neural implant. *Nature* 444 56–60. 10.1038/nature05226 17057705

[B9] Jackson A. Moritz C. T. Mavoori J. Lucas T. H. Fetz E. E. (2006b). The Neurochip BCI: towards a neural prosthesis for upper limb function. *IEEE Trans. Neural. Syst. Rehabil. Eng.* 14 187–190. 10.1109/tnsre.2006.875547 16792290

[B10] Jimbo Y. Kasai N. Torimitsu K. Tateno T. Robinson H. P. (2003). A system for MEA-based multisite stimulation. *IEEE Trans. Biomed. Eng.* 50 241–248. 10.1109/tbme.2002.805470 12665038

[B11] Liu X. Zhang M. Richardson A. G. Lucas T. H. Van der Spiegel J. (2017). Design of a Closed-Loop, Bidirectional Brain Machine Interface System With Energy Efficient Neural Feature Extraction and PID Control. *IEEE Trans. Biomed. Circuits Syst.* 11 729–742. 10.1109/tbcas.2016.2622738 28029630

[B12] Lucas T. H. Fetz E. E. (2013). Myo-cortical crossed feedback reorganizes primate motor cortex output. *J. Neurosci.* 33 5261–5274. 10.1523/jneurosci.4683-12.2013 23516291 PMC3684433

[B13] Mavoori J. Jackson A. Diorio C. Fetz E. (2005). An autonomous implantable computer for neural recording and stimulation in unrestrained primates. *J. Neurosci. Methods* 148 71–77. 10.1016/j.jneumeth.2005.04.017 16102841

[B14] McPherson J. G. Miller R. R. Perlmutter S. I. (2015). Targeted, activity-dependent spinal stimulation produces long-lasting motor recovery in chronic cervical spinal cord injury. *Proc. Natl. Acad. Sci. U. S. A.* 112 12193–12198. 10.1073/pnas.1505383112 26371306 PMC4593107

[B15] Mirbozorgi S. A. Bahrami H. Sawan M. Rusch L. A. Gosselin B. (2016). A Single-Chip Full-Duplex High Speed Transceiver for Multi-Site Stimulating and Recording Neural Implants. *IEEE Trans. Biomed. Circuits Syst.* 10 643–653. 10.1109/tbcas.2015.2466592 26469635

[B16] Moritz C. T. Perlmutter S. I. Fetz E. E. (2008). Direct control of paralysed muscles by cortical neurons. *Nature* 456 639–642. 10.1038/nature07418 18923392 PMC3159518

[B17] Newman J. P. Zeller-Townson R. Fong M. F. Arcot Desai S. Gross R. E. Potter S. M. (2012). Closed-Loop, Multichannel Experimentation Using the Open-Source NeuroRighter Electrophysiology Platform. *Front. Neural. Circuits* 6:98. 10.3389/fncir.2012.00098 23346047 PMC3548271

[B18] Nishimura Y. Perlmutter S. I. Eaton R. W. Fetz E. E. (2013). Spike-timing-dependent plasticity in primate corticospinal connections induced during free behavior. *Neuron* 80 1301–1309. 10.1016/j.neuron.2013.08.028 24210907 PMC4079851

[B19] Olsson R. H. III Buhl D. L. Sirota A. M. Buzsaki G. Wise K. D. (2005). Band-tunable and multiplexed integrated circuits for simultaneous recording and stimulation with microelectrode arrays. *IEEE Trans. Biomed. Eng.* 52 1303–1311. 10.1109/tbme.2005.847540 16041994

[B20] Rembado I. Song W. Su D. K. Levari A. Shupe L. E. Perlmutter S. I. (2021). Cortical responses to vagus nerve stimulation are modulated by brain state in non-human primates. *Cereb. Cortex* 1–19. 10.1093/cercor/bhab158 34151377 PMC8567998

[B21] Rembado I. Zanos S. Fetz E. E. (2017). Cycle-Triggered Cortical Stimulation during Slow Wave Sleep Facilitates Learning a BMI Task: a Case Report in a Non-Human Primate. *Front. Behav. Neurosci.* 11:59. 10.3389/fnbeh.2017.00059 28450831 PMC5390033

[B22] Richardson A. G. Fetz E. E. (2012). Brain state-dependence of electrically evoked potentials monitored with head-mounted electronics. *IEEE Trans. Neural. Syst. Rehabil. Eng.* 20 756–761. 10.1109/tnsre.2012.2204902 22801526 PMC6886250

[B23] Rizk M. Bossetti C. A. Jochum T. A. Callender S. H. Nicolelis M. A. Turner D. A. (2009). A fully implantable 96-channel neural data acquisition system. *J. Neural. Eng.* 6:026002. 10.1088/1741-2560/6/2/026002 PMC2680289 19255459

[B24] Rolston J. D. Gross R. E. Potter S. M. (2009a). A low-cost multielectrode system for data acquisition enabling real-time closed-loop processing with rapid recovery from stimulation artifacts. *Front. Neuroeng.* 2:12. 10.3389/neuro.16.012.2009 19668698 PMC2722905

[B25] Rolston J. D. Gross R. E. Potter S. M. (2009b). NeuroRighter: closed-loop multielectrode stimulation and recording for freely moving animals and cell cultures. *Conf. Proc. IEEE Eng. Med. Biol. Soc.* 2009 6489–6492. 10.1109/IEMBS.2009.5333589 19964440

[B26] Rosenthal J. Reynolds M. S. (2019). A 1.0-Mb/s 198-pJ/bit Bluetooth Low-Energy Compatible Single Sideband Backscatter Uplink for the NeuroDisc Brain-Computer Interface. *IEEE Trans. Microw. Theory Tech.* 67 4015–4022. 10.1109/tmtt.2019.2938162

[B27] Seeman S. C. Mogen B. J. Fetz E. E. Perlmutter S. I. (2017). Paired Stimulation for Spike-Timing-Dependent Plasticity in Primate Sensorimotor Cortex. *J. Neurosci.* 37 1935–1949. 10.1523/jneurosci.2046-16.2017 28093479 PMC5320619

[B28] Shon A. Chu J.-U. Jung J. Kim H. Youn I. (2018). An Implantable Wireless Neural Interface System for Simultaneous Recording and Stimulation of Peripheral Nerve with a Single Cuff Electrode. *Sensors* 18:1. 10.3390/s18010001 29267230 PMC5795569

[B29] Shupe L. Fetz E. (2021). An Integrate-and-Fire Spiking Neural Network Model Simulating Artificially Induced Cortical Plasticity. *Eneuro* 8 ENEURO.0333–20.2021. 10.1155/2021/6623926 33632810 PMC7986529

[B30] Sodagar A. M. Wise K. D. Najafi K. (2007). A fully integrated mixed-signal neural processor for implantable multichannel cortical recording. *IEEE Trans. Biomed. Eng.* 54 1075–1088. 10.1109/tbme.2007.894986 17554826

[B31] Su Y. Routhu S. Moon K. S. Lee S. Q. Youm W. Ozturk Y. (2016). A Wireless 32-Channel Implantable Bidirectional Brain Machine Interface. *Sensors* 16:1582. 10.3390/s16101582 27669264 PMC5087371

[B32] Tehovnik E. J. Tolias A. S. Sultan F. Slocum W. M. Logothetis N. K. (2006). Direct and indirect activation of cortical neurons by electrical microstimulation. *J. Neurophysiol*. 96 512–521. 16835359 10.1152/jn.00126.2006

[B33] Uehlin J. P. Smith W. A. Pamula V. R. Perlmutter S. I. Rudell J. C. Sathe V. S. (2020). A 0.0023 mm (2)/ch. Delta-Encoded, Time-Division Multiplexed Mixed-Signal ECoG Recording Architecture With Stimulus Artifact Suppression. *IEEE Trans. Biomed. Circuits Syst.* 14 319–331. 10.1109/tbcas.2019.2963174 31902767 PMC9482074

[B34] Yin M. Borton D. A. Komar J. Agha N. Lu Y. Li H. (2014). Wireless neurosensor for full-spectrum electrophysiology recordings during free behavior. *Neuron* 84 1170–1182. 10.1016/j.neuron.2014.11.010 25482026

[B35] Zanos S. Richardson A. G. Shupe L. Miles F. P. Fetz E. E. (2011). The Neurochip-2: an autonomous head-fixed computer for recording and stimulating in freely behaving monkeys. *IEEE Trans. Neural. Syst. Rehabil. Eng.* 19 427–435. 10.1109/tnsre.2011.2158007 21632309 PMC3159515

[B36] Zhou A. Santacruz S. R. Johnson B. C. Alexandrov G. Moin A. Burghardt F. L. (2019). A wireless and artefact-free 128-channel neuromodulation device for closed-loop stimulation and recording in non-human primates. *Nat. Biomed. Eng.* 3 15–26. 10.1038/s41551-018-0323-x 30932068

